# Large Scale Calcium Imaging of the Cerebellar Vermis During Sensory Stimulus Unravels Two Response’s Components That Differ in Their Spatiotemporal Properties

**DOI:** 10.3389/fnsys.2019.00018

**Published:** 2019-05-08

**Authors:** Hananel Byk, Guo-Jen Huang, Yoichiro Iwakura, Yosef Yarom

**Affiliations:** ^1^Department of Neurobiology, Silberman Institute of Life Sciences and Edmond & Lily Safra Center for Brain Sciences (ELSC), The Hebrew University of Jerusalem, Jerusalem, Israel; ^2^Department and Graduate Institute of Biomedical Sciences, College of Medicine, Chang Gung University, Taoyuan, Taiwan; ^3^Center for Experimental Animal Models, Institute for Biomedical Sciences, Tokyo University of Science, Tokyo, Japan

**Keywords:** cerebellar cortex, calcium imaging, CXCR 4, climbing fiber, oscillation

## Abstract

The well documented precision of the cerebellar sagittal organization is commonly used to compose a comprehensive view on principles of cerebellar function. However, the physiological manifestation of this organization is either limited to information derived from single unit recordings or from imaging of a small group of closely located neurons. Here we used large scale imaging to monitor calcium concentration changes in the entire vermal area of folia V and VI in anesthetized mice. We found that the response to a strong auditory input or electrical shock to the tail area is composed of an early and a late component that differ in their spatiotemporal properties. The early component occurs throughout the scanned area whereas the late component reflects synchronous activation of Purkinje cells located along symmetric parasagittal bands that correspond well to sagittal band 2+ (Sugihara and Shinoda, 2004). Similar organization was found in the rigorously disorganized cerebellum of *Cxcr4* KO mice, suggesting that the sagittal organization is determined by the climbing fiber inputs to the cerebellar cortex. The responses for both stimuli are followed by a prolonged recovery period but the rate of recovery from auditory stimulus is much longer, reflecting a different site for the adapting process. We suggest that these sensory inputs, which are commonly used to evoke startle response, activate two sets of climbing fiber inputs that differ in their spatiotemporal properties and contribute to the motor organization and habituation of the startle response.

Significance Statement:

The ensemble activity of neurons in the brain is one of the current challenges of neuroscience. Here we use a fast and large-scale calcium imaging system to monitor ensemble activity in the cerebellar cortex following auditory stimuli or electric shocks to the tail. The system, which enables the detection of the response to a single trail, reveals the robustness of the functional organization of the olivo-cerebellar system in sagittal bands that is preserved in genetically induced disorganized cerebellar cortex. Furthermore, the response, which represents the activation of two sets of climbing fibers inputs, is followed by a prolonged recovery process that indicates the cerebellar involvement in startle response.

## Introduction

The sagittal organization of the cerebellar cortex (CX), which has been documented by anatomical (Sugihara and Shinoda, 2004), physiological (Sugihara et al., 2007) and molecular (Gravel et al., 1987) studies, is now well accepted. This organization, which appears as longitudinal bands, or zones, and may extend throughout the cerebellar cortex, was originally related to the connectivity matrix of the Purkinje neurons (PN). Thus, PNs within a zone are innervated by climbing fibers (CFs) which originated at a specific subdivision of the inferior olive nucleus (IO). PNs’ output impinges on closely related neurons located in the deep cerebellar nuclei (CN). Currently 11 symmetrically positioned zones have been identified (Voogd and Glickstein, 1998) of which five are located within the vermal area. The longitudinal organization, defined by the connectivity matrix, corresponds precisely with the pattern of longitudinal organization revealed by molecular markers such as zebrin (Leclerc et al., 1992; Voogd, 2014). Furthermore and as expected, the longitudinal organization is also supported by physiological studies where the climbing fiber responses of various sensory modalities were examined (Andersson and Oscarsson, 1978; Bloedel and Ebner, 1984; Pakan et al., 2011). This specific and highly preserved organization has led to the concept of the “cerebellar module” which postulates that the basic cerebellar computational process is performed by the closed loop between the IO, PN, and CN (Apps and Garwicz, 2005; Apps and Hawkes, 2009; Cerminara et al., 2015).

In this study we used large-scale imaging of calcium concentration changes to document the functional organization of cortical responses to two stimuli paradigms: an auditory stimulus and an electric stimulation of the tail.

Auditory signals transmitted via mossy fibers specifically to vermal areas have been documented extensively (Shofer and Nahvi, 1969; Aitkin and Boyd, 1975; Altman et al., 1976) mostly in studies involving eye-blink conditioning paradigms where auditory stimuli are used as the conditioned stimulus. On the other hand, climbing fiber activation by auditory input has been documented in the context of the acoustic startle response (Mortimer, 1973; Altman et al., 1976).

The cerebellar responses to electrical tail stimulations have been studied in the context of mapping the functional organization of the cerebellar cortex (Jorntell et al., 2000). Climbing fiber responses to tail stimulation occur after a delay of about 20 ms and are restricted to a sagittal band in the vermal area. Tail stimulation has also been used in fear-conditioned bradycardia (Kotajima et al., 2014) where the involvement of the cerebellum, specifically the olivo-cerebellar pathway, was demonstrated.

In this study we also examine cerebellar response in *Cxcr4* KO mice. The anatomy of the cerebellar cortex of *Cxcr4* KO mice, which has been described in detail (Huang et al., 2014), shows a complete disappearace of foliar organization that is likely to be due to inappropriate PN and granular cell migration and malformation of PN axons and dendrites. Furthermore, these abnormalities are associated with severe motor impairments expressed as poor coordination and balance performance in skilled motor tests.

Thus, examining the functional organization of this cerebellum should shed some light on the morpho-function relations, particularly on the factors affecting the organization of the climbing fiber input.

In this work, we found that both auditory stimuli and electric shock to the tail elicit a two-component, complex calcium response with different spatiotemporal properties. One of the components is organized in sagittal bands which are preserved in the *Cxcr4* KO mice. We show how these responses are related to climbing fiber firing and present their adaptive behavior. We propose that these stimuli activate two sets of climbing fiber inputs that differ in their spatiotemporal properties and contribute to the motor organization and habituation of the startle response.

## Materials and Methods

Fluorescence responses to auditory stimuli or electrical stimulations of the base of the tail were recorded in 27 mice. Electrophysiological responses of 20 cerebellar cortical neurons were recorded simultaneously with imaging, 17 were responsive. Eight additional cells were recorded in response to stimuli without imaging procedure, seven were responsive.

### Animals

All procedures used in the study adhere to guidelines approved by the Hebrew University of Jerusalem Animal Care Committee (#12005). The Hebrew University is an Association for Assessment and Accreditation of Laboratory Animal Care (AAALAC)-accredited institution. In this study we used twenty seven C57BL/6 (“wild-type,” WT) mice, 2–4 months old and five Cxcr4(flox/flox) mice that were developed on C57BL/6 strain (Chung et al., 2010) and Sox1-Cre mice (Takashima et al., 2007) (Acc. No. [CDB0525K]^1^). The Cxcr4 mice have been described previously and were genotyped accordingly. Sox1-Cre mice express Cre throughout the neural tube at E9.5 (Takashima et al., 2007). Mice lacking Cxcr4 in the CNS were generated by crossing mice harboring loxP sites flanking exon 2 of the Cxcr4 gene (Cxcr4 flox/flox) with Sox1-Cre mice.

### Immunohistochemistry

All sections for Aldolase C staining were cut at a thickness of 40 μm on a sliding microtome. Sections were mounted on SuperFrost slides and dried overnight. Subsequently, slides were incubated in 0.01 mol/L citric buffer for 40 min at 90°C, 3% H_2_O_2_ for 10 min, rinsed in PBS, and incubated overnight at room temperature in Aldolase C/Zebrin II antibody (1:1000, Santa Cruz). Next day, a standard IgG ABC kit (Vector Lab) procedure was used and the slides incubated for 5–10 min with a Sigma DAB tablet. Sections were then counterstained with cresyl violet and mounted with DPX.

### Surgery and Calcium Sensitive Dye Loading

Mice were initially anesthetized with isoflurane (2.5% induction and surgery, 1% maintenance, in 100% O_2_). A craniotomy of 2–5 mm diameter was made over folia V, VI and paravermal areas of the cerebellar cortex. Patch glass pipettes (5–7 MΩ) were filled with AM ester of Oregon Green 488 Bapta-1 (dissolved in DMSO plus 20% Pluronic F-127) and diluted in a solution containing (mM): 135 NaCl, 1.8 CaCl_2_, 5.4 KCl, 1 MgCl_2_, and 5 Hepes, to yield a concentration of ∼4–8 mM. The same solution is used to cover the craniotomy. Pipettes were inserted to 150–200 μm depth and a pressure of 20–70 PSI was applied for 2 min, 5–10 injection sites are used for each experiment. After the dye injections anesthesia was switched from isoflurane to chloralose (dissolved in saline solution; i.p. 20 mg/kg/h) that enables sensory evoked responses. Several studies have measured sensory evoked activity in the cerebellar cortex under chloralose anesthesia, as well as induced motor responses by stimulating the cerebral motor cortex (Snider and Stowell, 1944; Ben Taib et al., 2005; Ben Taib and Manto, 2013). ECG and breathing were monitored along the experiment to assess depth of anesthesia, together with leg pinching. Body temperature was continuously monitored and maintained at 36 degrees with a heating pad that was controlled by the deviation from the desired 36 degrees.

### Data Acquisition

To image Ca changes we used a MiCAM ULTIMA (SciMedia) imaging and data-acquisition system, which uses a CMOS (complementary metal-oxide-semiconductor) sensor with 100 × 100 imaging elements, combined with a MVX10 macro-zoom microscope (Olympus). The acquisition rate ranged between 100 and 400 Hz. Exposed cortex was illuminated using epi-illumination with led light (480 nm) and appropriate filters and dichroic mirror. A *post hoc* filter based on the ECG recordings was used to subtract changes in signal induced by heart beat movements. Patch glass pipettes (5–7 MΩ) were used for single unit recordings from the cerebellar cortex. Recordings were made using an AxoClamp 2A (Axon Instruments, Union City, CA, United States) amplifier and sampled by a National Instruments board at rates of either 10 or 20 kHz (after being low pass filtered at 3 or 10 kHz).

### Sensory Stimulation

White noise and pure sine tones of 100 ms length (3 ms ramp) with a maximum of 20 dB difference in amplitude were used as auditory stimulus [3–19 kHz, 90–120 dB (SPL)]. Electrical stimulations of the base of the tail (1 ms and up to 10 mA) were used as a second modality of sensory input, with 1–5 stimuli given in 100 ms. Stimulations of different types (auditory, tail stimulation at different amplitude and frequency) were given in random order with an interval of 7 s. Pairs of stimulations with intervals of 300 ms to 2 s were used to reveal adaptation processes, and different pairs were given with in-between pair intervals of 8.5 s.

### Data Analysis

Custom-written Matlab code was used for imaging analysis including heartbeat movement subtraction based on ECG data and was averaged by regions of interest and then low-pass filtered with a Kaiser filter. Filter parameters were dependent on the imaging acquisition rate. For acquisition at 400 Hz, passband was defined from 0 to 100 Hz, and stopband for frequencies above 150 Hz, 5% ripple and stopband attenuation of 40 dB. For 200 Hz acquisition, passband was defined up to 66.6 Hz and stopband above 100 Hz. The size of calcium responses was evaluated by comparing the fluorescence during 60 ms preceding stimulations and the maxima reached within 140 ms after stimulation. Size normalization for adaptation analysis was in relation to the average response to the same stimulus presented as 1st stimulus. ROIs were determined manually on a custom-made GUI that presented a video on which correlated areas were selected.

Single unit analysis was performed using wave_clus (Quiroga et al., 2004), a spike sorting toolbox. In the cases that only complex spikes were recorded the possibility of recording from multiple units cannot be excluded.

## Results

### The Spatio-Temporal Organization of the Response to Auditory Stimulus

**FIGURE 1:**
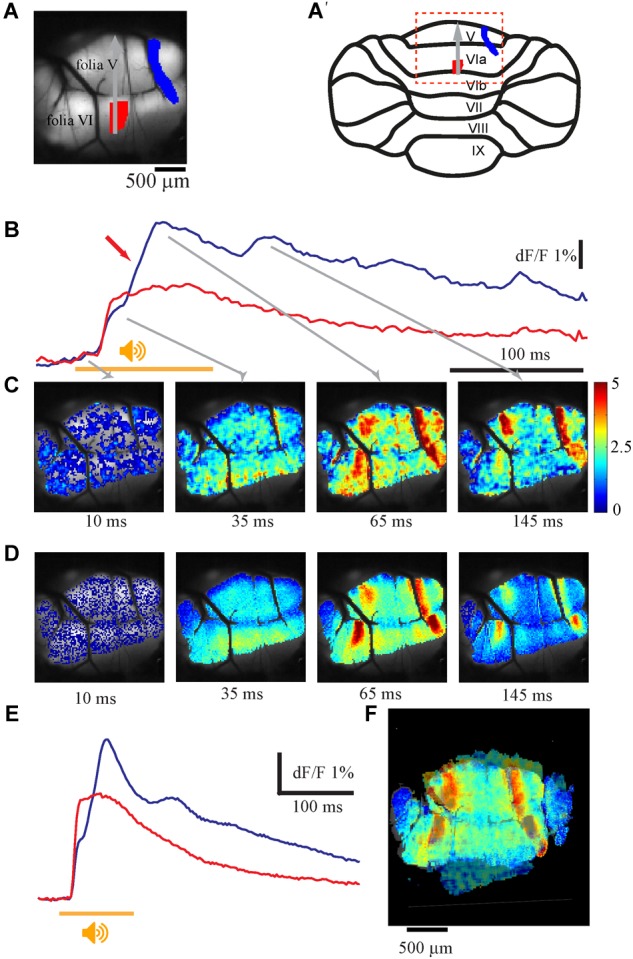
The auditory evoked response and its spatial distribution. **(A)** An anatomical view of the recorded area and its location in schematic illustration of the cerebellar cortex **(A′)**, red and blue area are the regions of interest for the traces shown in **B**. Gray arrow denotes the rostro-caudal axis at midline. Midline determined by the joint point between left and right side of the inferior colliculus above the vermis. **(B)** Fluorescence changes in two different areas evoked by pure tone of 12 kHz 110 dB (stimulus time indicated by an orange line), the 2nd calcium component marked with a red arrow. **(C)** The spatial distribution of the signal for the whole field of view at different time. The corresponding time for each frame is indicated by gray arrows in **B**. The intensity of the signal is color coded (bar, %). **(D,E)** The same as in **A** and **B** for an average response for 10 repeats. **(F)** Superposition of the spatial distribution of the peak response of the second component in four animals. The rostra-caudal and coronal axes of folia V were used as base for anatomical superposition. Note the two laterally positioned sagittaly organized bands (color is scaled differently for each experiment to highlight the lateral bands).

Complex fluorescence responses to auditory stimuli are readily obtained in the entire stained area (vermal and paravermal areas of folia V and VI). The complexity of the response manifests both in the temporal and spatial domains. In the temporal domain it is composed of two components; an initial component characterized by short delay to onset followed by a late response that occasionally demonstrates rhythmic-like activity. These two components differ in their spatial organization; while the first component usually appears in the entire stained area, the late response is restricted to well-defined sagittal bands. These properties are illustrated in Figure 1, where the scanning area is shown in Figure 1A and its location on schematic representation of the cerebellar cortex in Figure 1A′. The response to a single auditory stimulus (Figure 1B,C) and the average of 10 (Figure 1D,E), recorded at two regions of interest (ROI) shown in Figure 1A (matching colors). The spatial distribution of the response at 10, 35, 65, and 145 ms from stimulus onset is shown in Figure 1C (arrows indicate the temporal relation to responses in Figure 1B). The initial response started synchronously in both ROIs after a delay of 12.5 ms (average of 15.2 ± 1.2 ms, *n* = 27) from stimulus onset (orange bar) with a rise time of 15 ms (average 16 ± 3.2 ms, *n* = 27). The late response (marked by red arrow in Figure 1B), which peaks after a delay of 65 ms, was restricted to two laterally positioned and sagittally organized bands of 60–200 μm in width and extended length that crosses folia V and VI (Figure 1C 65 ms). The onset time of the late response varies across animals and ROIs from 35 up to 90 ms, while the rise time is highly dependent on the stimuli strength (15–60 ms). This complex spatio-temporal response to auditory stimuli was highly reproducible both in time and in different animals. Figure 1D,E show the average response to 10 stimuli, which is similar to the response of a single stimulus. Furthermore, the spatial distribution of the late response, manifested as two large lateral bands, in four animals are superimposed in Figure 1F, demonstrating significant overlap.

This late response often comprises multiple peaks at inter peak intervals of 80–110 ms, which correspond to a frequency of 11.53 +/-2.06 Hz and occur solely within the sagittal bands (Figure 1C,D). The number of peaks in the late response varies; four peaks can be distinguished in Figure 1B (blue trace); two peaks can be recognized in the average response in Figure 1E and on rare occasions up to nine peaks can be reached (see Figure 7). The rise time of each peak (15–60 ms) depends on the specific region and stimulus strength, with a larger response having a shorter rise time. The decay time after the second component has t_1/2_ of 190–220 ms. The response to auditory stimuli of different frequencies (3–20 kHz) and intensities shows similar tuning properties all over the vermis, with best frequencies varying between 9.5 and 15 kHz in different animals (Shofer et al., 1969) (data not shown).

### The Physiological Source of the Evoked Calcium Signal

**FIGURE 2:**
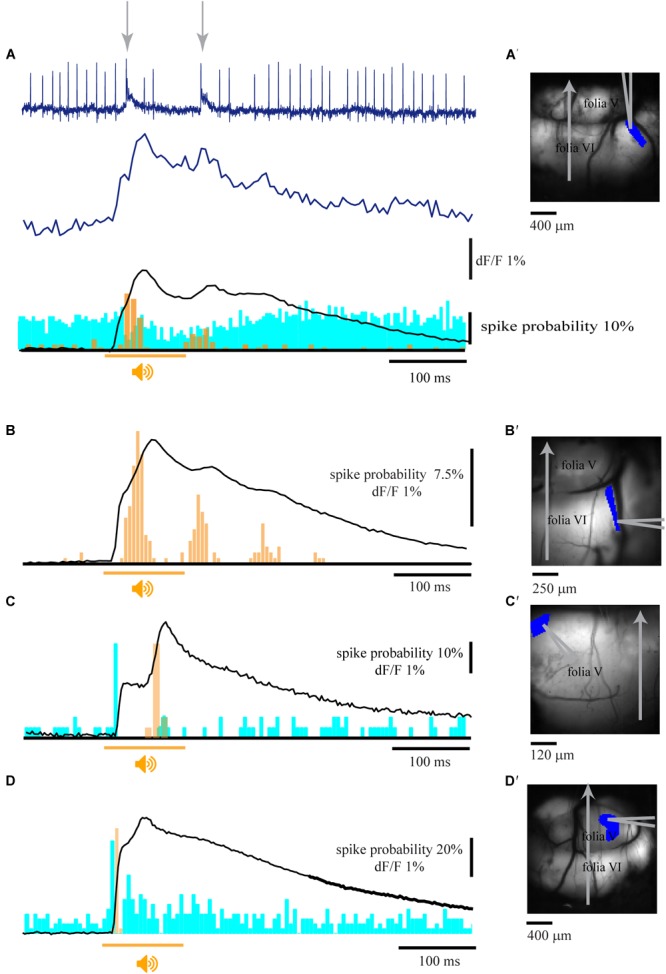
Climbing fibers are the main contributors to the fluorescence signal. **(A)** Extracellular recording from a single PN (top) and the corresponding florescence signal (Middle) during pure tone stimulation (9.5 kHz; 110 dB; arrows indicates the occurrence of complex spikes). Bottom – the average florescence response of 32 repeats and the corresponding complex spike (orange) and simple spikes (blue) PSTHs (96 trials). **(B)** The average florescence response of 180 trials and the corresponding PSTH histogram of CS activity, showing correlated multiple peaks in both the second florescence component and the PSTH. **(C)** Calcium signal and histograms of SS (blue) and CS (orange) for 30 trials of an auditory stimulus. Note a SS response at the beginning of the 1st calcium component and a CS response at the time of the 2nd calcium component. **(D)** Calcium signal and the corresponding activity histograms for 40 trials. Note that both SS and CS are correlated with the first calcium component. **(A′–D′)** the anatomical locations of the recordings in **A–D**, showing the corresponding ROIs and the location of the recording electrodes. Orange line represents the time of stimulus for all panels, CS histograms in orange and SS histogram in blue.

The electrophysiological correlates of the calcium signal were assessed by combining imaging and single unit recordings from cerebellar PNs (Figure 2). The activity of a single unit located within the imaging area (Figure 2A′) was measured with a patch electrode. Complex spikes (CS, arrows in Figure 2A top) are readily distinguished from simple spikes (SS) and highly correlated with the corresponding imaging trace from a region of interest determined by the location of the recording electrode (Figure 2A middle). The average imaging trace (Figure 2A bottom) was then correlated with the PSTH of the CS (orange) and SS (blue). The temporal correlation between CS activity and the fluorescence response was observed in 12 experiments of which in six cases CSs were highly correlated with the late component, reaching a peak that corresponded to the rise time of the fluorescence signal (Figure 2A bottom,B,C) and accurately followed the periodic feature of the late response (Figure 2A bottom,B). Only in three experiments an increase in CS activity was correlated with the early fluorescence signal (Figure 2D) and in another three a decrease in CS was measured. It should be noted that the CS were correlated to either the early or the late response but never with both (see section “Discussion”).

In 11 experiments where the SS activity was modulated by the auditory stimulus, seven neurons showed an increase in firing correlated with the early component with a peak at 12–18 ms from stimulus onset (Figure 2C,D) whereas six neurons displayed late decrease in activity (Figure 2A,C).

In view of these results we concluded that climbing fiber activation is involved in both early and late fluorescence responses. However, whereas the climbing fibers are the exclusive source of the late component, contribution of mossy fibers to the early component cannot be excluded.

### Spontaneous Calcium Signals Are Organized in Sagittal Bands and Correlated With CS Activity

**FIGURE 3:**
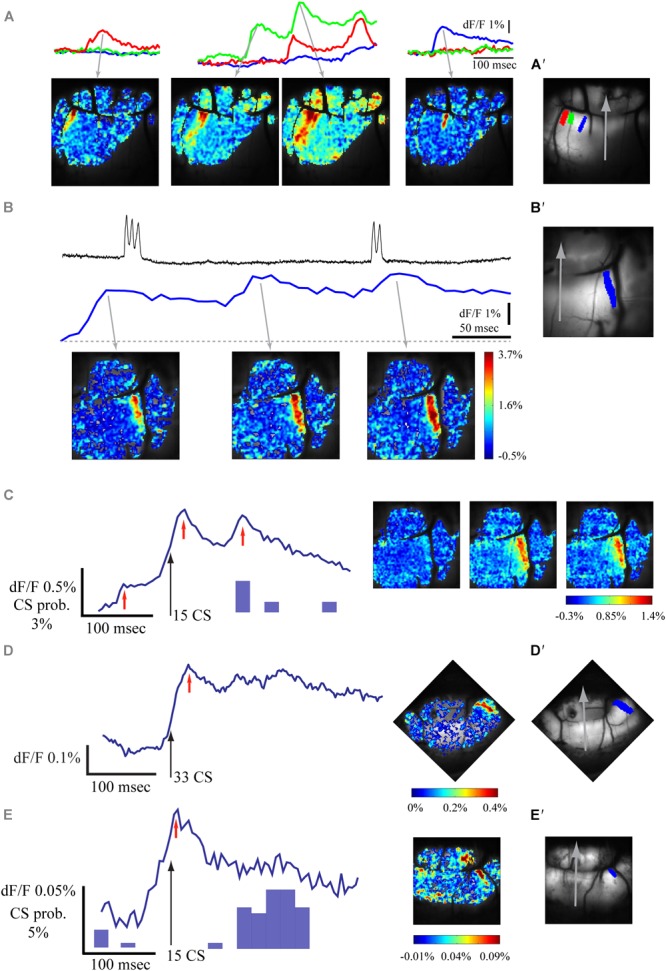
Spontaneous fluorescence responses are also correlated with climbing fibers activity. **(A)** Examples of spontaneous calcium transients in different sagittal bands. Top – calcium signal in three regions of interest marked with corresponding color in **A′**. Bottom – spatial distribution of the signal at the peaks of the fluorescence (gray arrows). **(B)** An example of spontaneous complex spikes (upper trace) recorded simultaneously with the calcium signal (middle and bottom). Note that the CS are correlated with only two of three peaks of the calcium signal. **(C)** The average signal aligned on spontaneous CS events (left, black arrow; for the same experiment as in **B**) and its spatial distribution (right) at the time denoted by red arrows. **(D,E)** Two examples of fluorescence measurements, aligned on CS (as in **C**) Note the sagittal organization in the spatial distribution images (in **D** the sagittal organization is along the paravermal areas). In **C** and **E**, the histogram of the CSs is shown (in **D**, all relevant CS were aligned on the black arrow). **(A′,B′,D′,E′)** Anatomical views of the recorded field of view in **A, B + C, D**, and **E**, respectively, a gray arrow indicates the medial sagittal axis, the regions of interest for traces are colored.

A low rate of spontaneous fluorescence activity was often encountered. Examples are shown in Figure 3A where the spontaneous activity was measured in 3 ROIs (Figure 3A′); their corresponding spatial distribution is shown in the lower panel. This activity has the characteristics of the late evoked response described above. The rise time of these events varies between 15 and 60 ms and up to 4% change in fluorescence was measured. Occasionally the spontaneous events, which are organized in characteristic sagittal bands (Figure 3A′,B′), show multiple peaks at an interval of 80–110 ms (Figure 3A-middle,B). These features of the spontaneous activity suggest that they represent the occurrence of spontaneous PN complex spikes. Indeed, measuring the unit activity (Figure 3B upper trace) simultaneously with the change in fluorescence (Figure 3B middle trace) suggests close correlation. Furthermore, in this example (Figure 3B) the spontaneous calcium signal is composed of three events while the unit recording shows only two events, suggesting that more than one PN is involved with the generation of the spontaneous calcium signal. Moreover, aligning the fluorescence recordings with the CS occurrence (Figure 3C), revealed a sagittally organized (Figure 3C right) peak in fluorescence signal (Figure 3C left). The peak response was followed by an additional peak, occurring at a delay of 80 ms that was correlated with a peak in the CS histogram. It should be noted that the small calcium signal that occurs 80 ms before the aligned CSs is not correlated to the CSs histogram, again suggesting the participation of other cells in the correlated activity. Another two examples of this analysis are shown in Figure 3D,E, revealing calcium transients organized in parasagittal bands. It should be mentioned that the amplitude of these averaged fluorescence signals is 5–20 times smaller than the late component of the auditory evoked responses, suggesting that the number of PNs participating in spontaneous activity is smaller than the number of PNs participating in evoked responses.

### The Sagittal Organization of the Auditory Response Is Preserved in *Cxcr4* KO Mice

The robustness of the sagittal organization of the auditory response was examined in *Cxcr4* KO mice, where severe deformation of cerebellar morphology has been documented.

**FIGURE 4:**
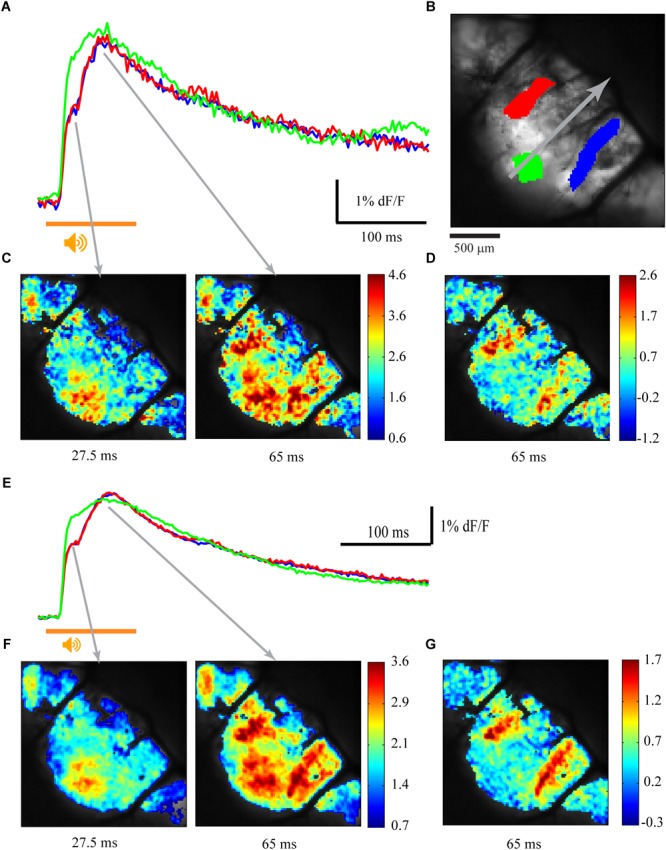
Sagittal organization of the response is preserved in *Cxcr4* KO mice. **(A)** Calcium responses to auditory white noise stimulation (orange line) in three regions of interest. **(B)** The anatomical map, with three regions of interest marked in colors, corresponding to traces in **A** and **E**. The gray arrow indicates the midline of the rostro-caudal axis (folia V, VI, and paravermis are visible). **(C)** The spatial distribution of the fluorescence signal relative to baseline calcium signal (%) at 27.5 and 65 ms after stimulus onset (gray arrows mark the temporal relation to the records in **A**). **(D)** The signal at 65 ms after stimulus onset relative to end of 1st calcium component (27.5 ms). **(E,F,G)** The same as in **A, C**, and **D** for an average signal of 10 trials.

**FIGURE 5:**
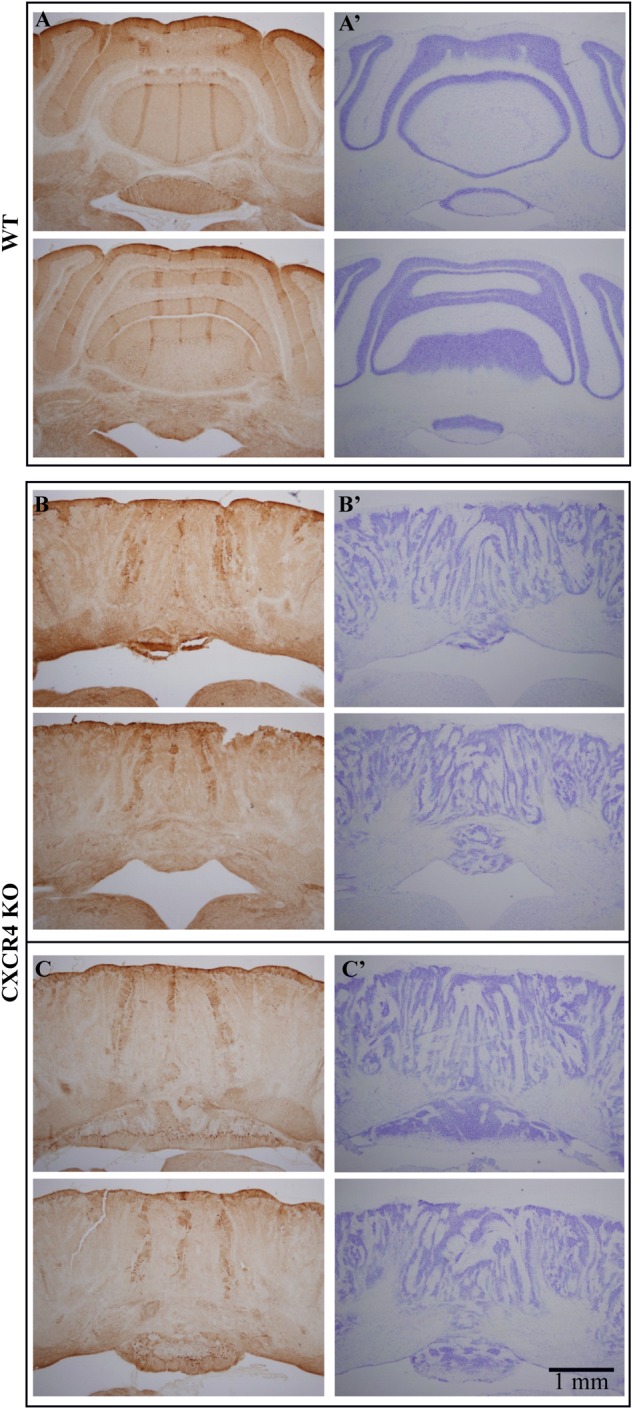
Zebrin labeling of the cerebellum of *Cxcr4* KO mice supports the sagittal organization. Coronal sections stained for zebrin (Left panels) and Nissl (right panels), each sample shows two levels along the rostro-caudal axis. **(A,A′)** WT animal **(B,B′)** *Cxcr4* KO animal. **(C,C′)** the same as **B** and **B′** for another KO animal. Note the three zebrin bands that appear in both, WT and *Cxcr4* KO mice, while the cellular staining reveals a complete cortical disorder in KO animals.

The response of the cerebellar cortex of *Cxcr4* KO mouse to auditory stimuli is shown in Figure 4. The absence of foliar organization is demonstrated in Figure 4B (compare to Figure 1, 2, 3 where the anatomical border between folia V and VI are clearly seen; see also Figure 5). The response to auditory stimulus clearly shows the same two components as in a wild type animal (Figure 4A and averaged over 10 repeats in Figure 4E): an early spatial non-specific activation followed by a late sagittally organized component (red and blue traces). The sagittal organization of the second component is better illustrated by setting the time of the end of 1st component (27.5 ms) as fluorescence baseline for a single trial (Figure 4D) and for an average of 10 (Figure 4G). This laterally positioned sagittal organization, which is very similar to the results for a WT animal (Figure 1), suggests that the modular organization of IO projection to cerebellar cortex is maintained in *Cxcr4* KO mice. Although the comparison was not performed between littermates, which might question the validity of our conclusions (see discussion), the zebrin labeling that was accurately performed shows that in the WT the distribution is essentially identical to what has been described (see an example in Abbott et al., 1996).

Following the physiological results, we searched for molecular support to the preservation of sagittal organization. To that end, we performed zebrin labeling in *Cxcr4* KO mice and compared the results to those obtained in WT mice. Figure 5 shows cross sections of the cerebellum from WT (top) and two mutant mice (bottom), each at two coronal levels, stained with Nissl (right) and zebrin II (left). In accordance with previous reports, sagittal bands of zebrin labeling are clearly seen (Figure 5A, left) in the well-organized cerebellum (Figure 5A, right) of the WT mice. Surprisingly, sagittal bands of zebrin labeling can also be clearly recognized in the mutant mice (Figure 5C,D, left) although the crystalline organization of the cerebellum is totally lost (Figure 5B,C, right). Furthermore, the two lateral zebrin bands fit the location of the two fluorescent bands observed in response to auditory stimuli, suggesting that the CF inputs determine the sagittal bands (see discussion).

### Repeated Sensory Stimulation Unravels a Robust Adaptive Process

**FIGURE 6:**
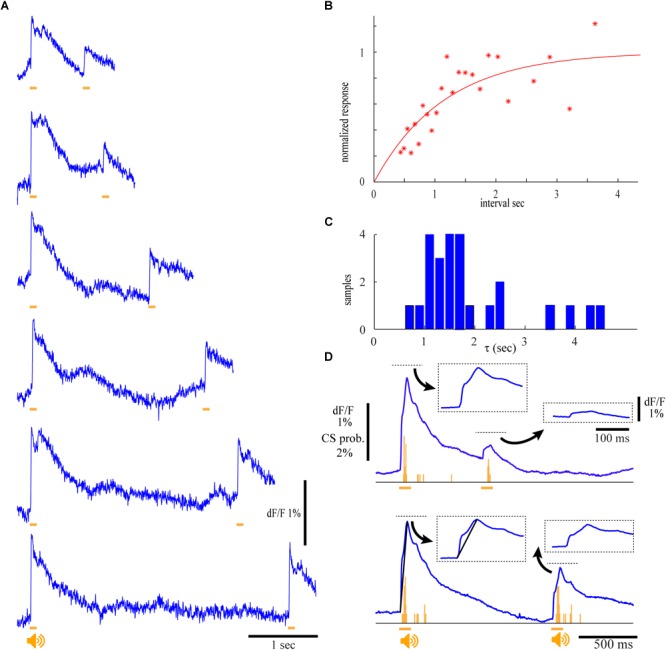
The auditory response undergoes pronounce and long lasting adaptation. **(A)** Calcium traces for two identical auditory stimuli (9.5 kHz, 120 dB) delivered at different inter stimuli interval. Note that the recovery of the 2nd response occur only after long intervals. **(B)** The normalized calcium response of the second auditory stimulus as a function of the inter stimulus interval. Red line denotes the fitting of exponential function with a time constant (τ) of 1.15 s (*r*^2^ = 0.64). **(C)** The distribution of τ in four experiments. The responses in each experiment were analyzed in eight ROIs and two different auditory stimuli were used. An average value of 1.93 sec was calculated for 25 out of 32 experiments where fitted *r*^2^ values were above 0.3. **(D)** The average calcium responses of 10 repeats at two intervals and the corresponding histograms of CS activity. The four responses (marked by dashed rectangles) are shown at higher temporal resolution.

As mentioned above, activation of climbing fibers by loud auditory stimuli has been studied in relation to the acoustic startle response which is known to undergo pronounced and prolonged adaptive process. Therefore, we studied the auditory responses to a pair of stimuli delivered at different intervals (Figure 6). At intervals shorter than 600 ms (Figure 6A, upper trace) the response to the second stimulus was dramatically reduced to less than 25% of the first response, followed by a slow recovery process which even after 2 s failed to reach full recovery. The recovery follows an exponential process (Figure 6B) with a time constant of 1.15 s (*r*^2^ = 0.64). On the population level the time constant of recovery varied between 1 and 4 s with an average of 1.5 s (Figure 6C). Furthermore, the reduction in the fluorescence response to the second stimulus was accompanied by a reduction in CS activity. This is demonstrated in Figure 6D where prolonged repetitions of two different intervals enabled not only the construction of the associated CS histograms (Figure 6D, orange bars) but also to dissociate between the early and late components of the fluorescence response (insets). Indeed, the decrease in the fluorescence response to the second stimulus is accompanied by a matching reduction in CS activity. Furthermore, the adaptive process affects the early and late components of the fluorescence response to a similar extent. While the amplitude of the first component of the response to the first stimulus was on average 0.9%, it was reduced to 0.15 and 0.45% for 600 and 1000 ms intervals. The average reduction of the second component was from 0.7 to 0.1% and 0.45% for the two intervals.

### Modality Comparison of Calcium Response

Paired pulse depression of the CF synaptic current, which has been thoroughly studied, demonstrates an exponential recovery process with time constants of a few seconds (Hashimoto and Kano, 1998). Despite the apparent similarity to the adaptive process described above, it is difficult to extrapolate from synaptic currents to changes in calcium concentration measured in our experiments. However, if the source of the adaptive process is within the olivo-cerebellar loop, it should be independent of the sensory input that activates the cerebellar cortex. We examined this possibility by electrical stimulation of the base of the tail (see section “Materials and Methods”). Such stimulation is likely to activate spino-cerebellar tracts that convey sensory information on muscle length and tension, although pain related information cannot be excluded.

**FIGURE 7:**
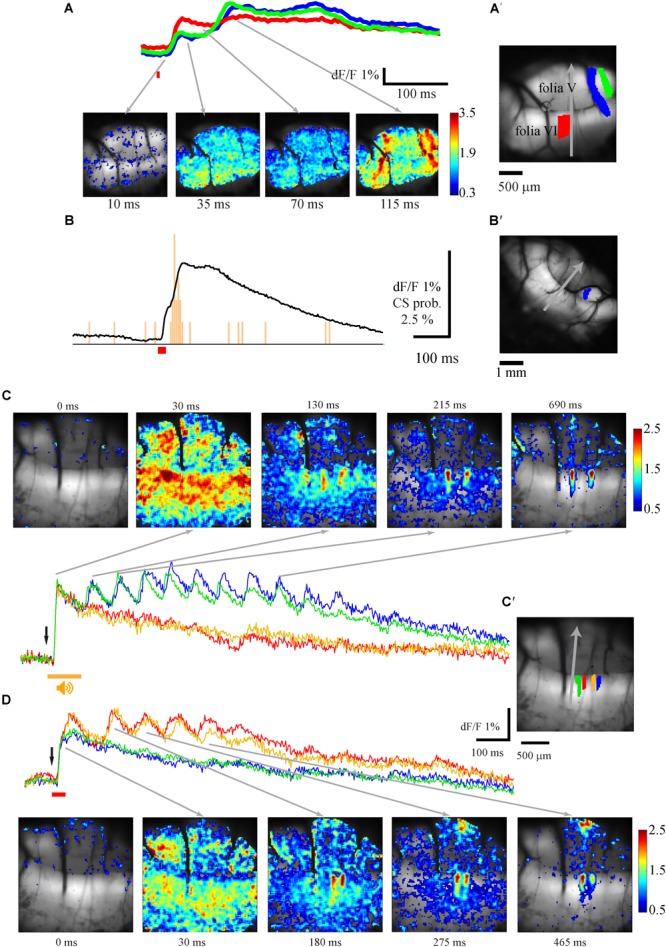
The spatio-temporal organization of the response to electric stimulation of the tail. **(A)** The spatio-temporal properties of the response to stimulation of the animal tail (time of stimulation is indicated by red bar). Top – the temporal properties of the responses measured at three ROIs shown, in matching color, in **A′**. Bottom – The spatial distribution of the response at different time. The time of each frame is indicated below and the relation to the temporal description is indicated by arrows. **(B)** The average response to tail stimulations recorded from the ROI shown in **B′** and the histogram of CS activity recorded simultaneously from PN. **(C,D)** the spatio-temporal properties of the response to auditory **(C)** and tail **(D)** stimulation in the same animal in four ROIs shown in **C′** with matching colors. **C** top and **D** bottom – the spatial distribution of the responses at different times from stimulus onset. Gray arrows mark the temporal relation to the records. Response size is color scaled in %.

The fluorescence responses to such a stimulus are shown in Figure 7. The average response (Figure 7A upper panel) at the specific regions of interests (Figure 7A′, matching colors) and the corresponding spatial distribution (Figure 7A lower panel) show that as with the auditory stimuli, the response is composed of two components: a broadly distributed early component, which appears after a delay of 12.5 (12.5–17.5 ms) from stimulus onset and reaches a peak after 20 ms (20–30 ms), followed by a second component which slowly decays over time course of few seconds. The late component, which is spatially organized in specific sagittal bands (Figure 7A bottom, 115 ms), tends to display multiple peaks (see also Figure 7D). Similar to the auditory response, CSs are the exclusive source of the late component, as evident from the CS histogram of a PN that was simultaneously measured with the calcium signal (Figure 7B). For detailed comparison of the spatial distributions of the responses to the different stimuli we measured the responses to auditory (Figure 7C) and tail (Figure 7D) stimuli in the same animal. In this particular example a prolonged period of rhythmic activity with a very similar frequency of ∼13 Hz was triggered by both stimuli. Although the spatial distribution of the responses of both stimuli is rather similar (Figure 7C,D), close examination revealed spatial differences that are best shown by comparing the regions of interest (Figure 7C′). Whereas the blue and green regions were activated only by the auditory stimulation, the orange and red regions were activated by the tail stimulations. Such a complete spatial separation between modalities during the 2nd component was rarely encountered, although differences in the sensitivity were usually observed. Thus, although both stimuli elicited similar responses, their late components differed in their spatial organization.

### Cross-Modal Adaptive Process

**FIGURE 8:**
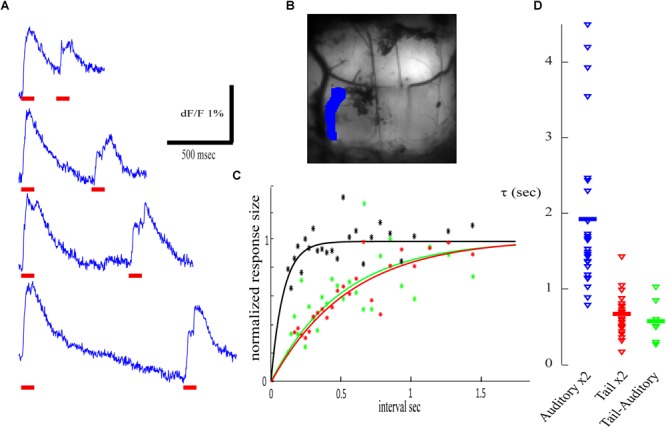
Cross modal stimulation and adaptation. **(A)** Calcium traces for two identical electric stimuli to the tail (marked by red horizontal bars) delivered at different inter stimuli interval. **(B)** An anatomical view of the recorded area and the corresponding ROI. **(C)** The normalized calcium response of the second stimulus as a function of the inter stimulus interval fitted by exponential function. Red- paired tail stimuli (τ) of 0.56 s (*r*^2^ = 0.80); green- tail followed by auditory stimulus (τ = 0.50 s, *r*^2^ = 0.47); black- auditory followed by tail stimulus. **(D)** The distribution of τ in four experiments. The responses in each experiment were analyzed in eight ROIs and in four combinations, only values with *R* square above 0.3 are presented: auditory – auditory (blue; *R*^2^ > 0.3 for 78.125%, average 1.927 s) from Figure 7A; tail – tail (red; *R*^2^ > 0.3 for 68.75%, average 0.675 s); auditory – tail (*R*^2^ > 0.3 for 21.875%, average 0.580 s). No significant effect was detected in tail- auditory combination (black in **C**, no significant τ to show in **D**).

The paired pulse paradigm of tail stimulation revealed that, as for the auditory response, a pronounced reduction (up to 80%) followed by a slow exponential process of recovery was always observed (Figure 8A, recorded from the blue ROI in Figure 8B; compare to Figure 6A). However, the time constant of the recovery process was below 1 s. In the presented example (Figure 8C, red) a time constant of 0.56 s. (*r*^2^ = 0.8) was calculated and an average of 0.675 s was calculated for *n* = 22 (Figure 8D, red).

Next, we examined cross-modal interactions where tail stimulation was preceded by an auditory stimulus and vise-versa. Unfortunately, we could not find areas with complete modality separation in cross-modal interactions experiments. When an auditory stimulus was preceded by an electric stimulation of the tail (Figure 8C, green), an exponential recovery process with a time constant similar to paired tail stimulations (0.51 s; *r*^2^ = 0.47) was found. However, in reverse order, auditory stimulation failed to induce depression of the tail response (Figure 8C, black *r*^2^ < 0.3). It should be noted that the shortest intervals used in these experiments were 180 ms and thus short-lasting depression cannot be excluded. These results are summarized in Figure 8D, comparing the distribution of the calculated time constant for the recovery process of paired auditory stimulation (blue), paired tail stimulation (red) and that of cross-modal interaction (green) where the auditory stimulus was preceded by an electric stimulation of the tail. These cross-modal interactions raise some questions on the neuronal site that produces the paired pulse depression (see discussion).

## Discussion

This study is focused on the spatio-temporal organization of responses in the cerebellar cortex to strong sensory stimuli that in an awake animal would be regarded as aversive stimuli. To this end we used a large-scale imaging system that enables screening for calcium changes in the entire folia V and VI of the vermal area, demonstrating the robustness of the cerebellar sagittal organization.

### The Physiological Source of the Calcium Signal and Its Relation to the Startle Response

It is commonly accepted that the main contributor to calcium signals in the cerebellar cortex are the PN complex spikes evoked by CF activity. Indeed, we showed that CSs are correlated with both components of the calcium signals that differ in their spatiotemporal properties. The first component, which appears at narrow range of delays from stimulus onset 12–17.5 ms, occurs all over the recording area whereas the second component, which appears at a more variable delay (30–110 ms), occurs at restricted sagittal bands and is usually associated with rhythmic activity. This sagittal organization is in agreement with recent studies showing a spatial correlation between complex spike synchrony and aldolase C compartments (Tsutsumi et al., 2015) as well as with previous anatomical work of Sugihara and colleagues (Sugihara and Shinoda, 2004). Accordingly, our lateral sagittal bands, which are shown in Figure 1C,D,F, are compartment number 2+ (Sugihara and Shinoda, 2004) whose CF input originates in the principal olive. The source of the first component of the calcium signal is somewhat more difficult to identify. We demonstrated that CSs in a given PN were either correlated with the first or the second component but never with both, suggesting two different populations of CFs. On the other hand, SS activity was correlated only with the first component. There is no question that mossy fibers are activated during auditory or tail stimuli and thus raise the possibility that some of the responses do not represent PN activity. However, the large Ca signals measured in our experiments suggest that our system detects mostly CF responses.

Thus, we concluded that the recorded calcium signal reflects the activation of two different networks of CFs that activate the cerebellar cortex to different extent in the temporal and spatial domains. It is therefore tempting to speculate that the sensory stimulation triggers a large population of IO neurons that can be subdivided into oscillating and non-oscillating neurons. The non-oscillating neurons respond with a short delay (1st calcium component) with a narrow range of variability. In oscillating neurons, which correlate exclusively with the 2nd calcium component, the input will sum with the subthreshold oscillation activating the neurons with a rather long and variable delay and will be followed by rhythmic responses. One would expect that in such a scenario where the time of input is uncorrelated with the subthreshold oscillations, repeating the same stimulus will lead to different delays; however, our results do not support this expectation. Nevertheless, our previous work demonstrated that synaptic input can reset the subthreshold activity (Lefler et al., 2013) thereby imposing similar delays upon repeating input and generating rhythmic activity that is preserved upon averaging.

Finally, the complex spatiotemporal response of the cerebellar cortex can contribute to the kinetics of the behavioral response to aversive stimuli known as the “startle response” (Koch, 1999). This response is characterized as an all-body muscle spasm followed by a short period of muscle tremor (Grosse and Brown, 2003). The rhythmic behavior of the second component of the calcium response can well be correlated with the delayed tremor of the startle response. The involvement of the olivo-cerebellar system in tremor generation is well documented (Llinas and Volkind, 1973). However, studies of the startle response suggest that the olivo-cerebellar system is involved in adaptation of the startle response (see below) but not in the induction of the response (Desperati et al., 1989; Lopiano et al., 1990). Except of the work of Gruart et al. (1995) where air puffs evoked two successive bursts, in line with our observations, most of these studies examine mostly the initial “jerked movement” without detailed examination of the response kinetics.

### Spontaneous Activity

We show that the spontaneous calcium events are also organized in parasagittal bands. From the spatial organization of these events one can deduce that they reflect a synchronous activation of CFs. The occasional appearance of spontaneous rhythmic activity as well as the correlation with spontaneous CS further supports this possibility. It should be noted that the spontaneous activity is limited to the sagittal bands. Since it is unlikely that our imaging system can detect the CS activity of a single PN, we must assume that the middle area is innervated by CFs that are not synchronously active. This possibility is in line with the above suggestion that we are looking at two different populations of olivary neurons. One population includes neurons that generate subthreshold oscillations that innervate specific cortical areas organized in sagittal bands. Thus, there is a high probability for spontaneous synchronized activity in a group of neurons. The second population includes non-oscillating olivary neurons and therefore the probability of spontaneous activity of a group of neurons is low. This second group innervates the middle vermal areas and therefore this area is devoid of spontaneous calcium signals. Having said that, one should bear in mind that our experiments were performed in anesthetized animals and the existence of subthreshold oscillations in awake conditions is still argued.

### *Cxcr4* KO Mice and Olivo-Cerebellar Mapping Development

Cerebellar and motor abnormalities in *Cxcr4* KO mice have been described in detail (Huang et al., 2014). Therefore, it is rather surprising that the sagittal organization is preserved. Furthermore, the zebrin labeling shows PN cell bodies stained along sagittal bands, preserving the olivo-cerebellar mapping. These surprising results, which strongly support the close link between zebrin labeling and physiological organization of CFs input, touch upon basic questions regarding the role of climbing fibers in cerebellar development and organization.

Two main mechanisms have been proposed as the source for olivo-cerebellar mapping. Sotelo and collaborators postulated that the cerebellum and the inferior olive might have matching gene expression domains that establish bidirectional signaling to generate the olivo-cerebellar map (Sotelo and Wassef, 1991; Sotelo and Chedotal, 1997, 2005). Support for this hypothesis was provided by a combination of markers that labeled zones of PNs (calbindin, GMP-cyclic dependent protein kinase, PN-specific glycoprotein, and PEP-19) and also marked corresponding subsets of inferior olive cells along with their projections. The precision and reproducibility of zonal boundaries defined by these markers suggested the possibility that inferior olivary neurons might target PN zones by recognizing positional cues (Sotelo and Wassef, 1991; Sotelo and Chedotal, 1997, 2005). Our data does not support this possibility but rather suggests that the Zones are determined by the CFs that force the PN to express the specific markers.

### Sensory Adaptation

Both auditory and tail stimuli are commonly used to induce startle responses in awake animals. Furthermore, a reduction in the magnitude of the startle response can be induced by two consecutive auditory stimuli, known as “Pre-Pulse Inhibition” (PPI; Koch, 1999). The brain mechanisms underlying PPI are not completely understood. PPI is absent in mutant mice where a massive loss of PNs was reported (Porras-Garcia et al., 2005). On the other hand it has been proposed that inhibition through the pedunculopontine tegmental nucleus on the caudal pontine reticular nucleus drives a smaller excitation of the startle pathway (Koch, 1999). Here we demonstrated a long-lasting reduction in the calcium response to a second auditory stimuli, exhibiting an exponential recovery process with a time constant of about 2 s that is well within the range of PPI. Therefore, we would like to suggest that the olivo-cerebellar path is involved in the PPI, a possibility that is in agreement with the finding that PPI does not occur in the absence of PNs.

**FIGURE 9:**
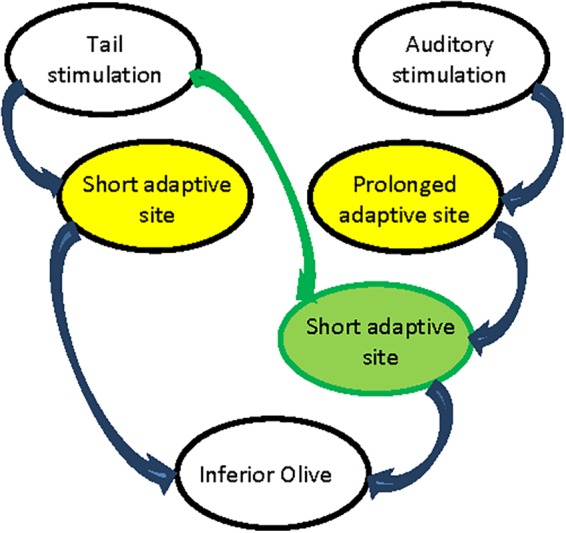
Schematic representation of adaptive process paths. Prolonged adaptive process sites in yellow. Short adaptive process sites in green.

Regardless of the involvement of the cerebellum in the adaptation of the startle response, we demonstrated two processes of reduction in the cerebellar responses to pairs of sensory stimulations. In both processes a significant reduction in CF input was observed and yet the recovery after tail stimulation is twice as fast as the recovery from auditory stimulation. Thus, we must conclude that there are two sites of adaptive process located upstream to the inferior olive (marked by yellow in Figure 9). Thus, the tail stimulation passes through a short adaptive site while the auditory input passes through a prolonged adaptive site. Moreover, the fast recovery of the auditory response that followed tail stimulation indicates that along the auditory path there is a second short adaptive site that is activated by the tail stimulation (Figure 9 green circle). According to this hypothetical arrangement the response to a pair of auditory inputs is determined by the prolonged adaptive site while the short adaptive site will be ineffective: the outcome of the fast recovering process is not visible due to the prolonged recovery upstream. Furthermore, the absence of an auditory activated adaptive site along the tail path can account for the ineffectiveness on tail response when preceded by auditory stimulation.

To conclude, we used a large field, fast imaging setup to monitor calcium signal in the cerebellar cortex during spontaneous and evoked activity. We demonstrate the robustness of the sagittal organization of the CF input evoked by an aversive-like stimulus and suggest that the pattern of the response originating from the IO can account for some of the startle response properties.

## Ethics Statement

All experimental procedures were approved by Hebrew University’s Animal Care and Use Committee.

## Author Contributions

HB and YY involved in imaging, electrophysiology, all analysis, and manuscript writing. G-JH and YI performed the zebrin labeling and provided the CXCR-4 mutant mice.

## Conflict of Interest Statement

The authors declare that the research was conducted in the absence of any commercial or financial relationships that could be construed as a potential conflict of interest.

## Footnotes

1 http://www.cdb.riken.jp/arg/mutant%20mice%20list.html
